# Neurotechnological Approaches to the Diagnosis and Treatment of Alzheimer’s Disease

**DOI:** 10.3389/fnins.2022.854992

**Published:** 2022-03-24

**Authors:** Shen Ning, Mehdi Jorfi, Shaun R. Patel, Doo Yeon Kim, Rudolph E. Tanzi

**Affiliations:** ^1^Genetics and Aging Research Unit, McCance Center for Brain Health, MassGeneral Institute for Neurodegenerative Disease, Department of Neurology, Massachusetts General Hospital and Harvard Medical School, Boston, MA, United States; ^2^Graduate Program for Neuroscience, Boston University School of Medicine, Boston, MA, United States; ^3^Center for Engineering in Medicine and Surgery, Department of Surgery, Massachusetts General Hospital and Harvard Medical School, Boston, MA, United States

**Keywords:** Alzheimer’s disease, neurotechnologies, diagnosis, therapeutic, amyloid

## Abstract

Alzheimer’s disease (AD) is the most common cause of dementia in the elderly, clinically defined by progressive cognitive decline and pathologically, by brain atrophy, neuroinflammation, and accumulation of extracellular amyloid plaques and intracellular neurofibrillary tangles. Neurotechnological approaches, including optogenetics and deep brain stimulation, have exploded as new tools for not only the study of the brain but also for application in the treatment of neurological diseases. Here, we review the current state of AD therapeutics and recent advancements in both invasive and non-invasive neurotechnologies that can be used to ameliorate AD pathology, including neurostimulation via optogenetics, photobiomodulation, electrical stimulation, ultrasound stimulation, and magnetic neurostimulation, as well as nanotechnologies employing nanovectors, magnetic nanoparticles, and quantum dots. We also discuss the current challenges in developing these neurotechnological tools and the prospects for implementing them in the treatment of AD and other neurodegenerative diseases.

## Introduction

**FIGURE 1:**
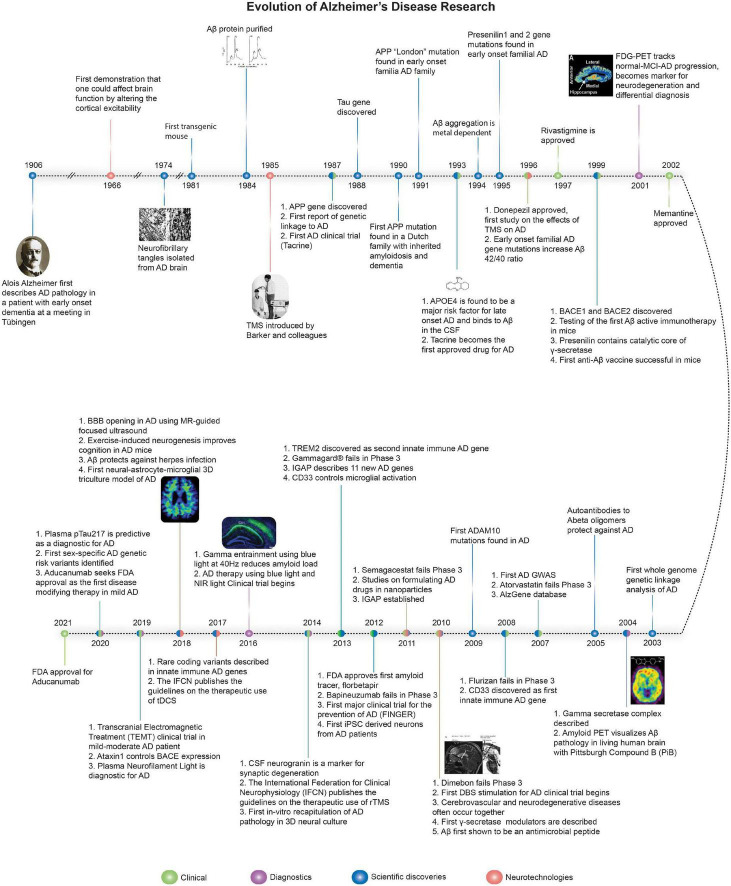
The evolution of Alzheimer’s disease. Timeline of major discoveries, technologies, therapeutics, diagnostics, and clinical trials for AD. Sources for images used in Figure 1 are available in the Supplementary Material.

**FIGURE 2:**
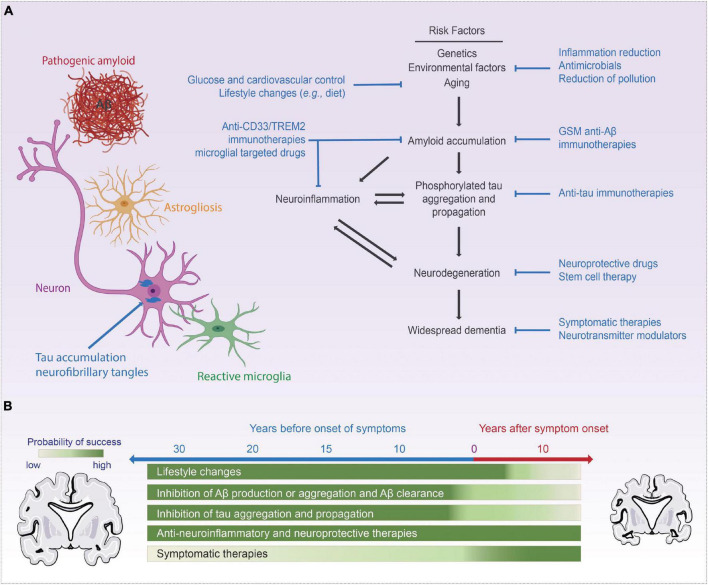
Current AD treatments and targets. Panel **(A)** commonly accepted AD disease progression pathway while highlighting commonly targeted pathways in AD therapeutics, **(B)** visualization of the time point those therapies are tackling in respect to the progression of the disease. Portions of the figure was created with BioRender.com.

Alzheimer’s disease (AD) is clinically defined by progressive cognitive decline and memory loss. The three key pathological hallmarks of AD include: (i) extracellular β-amyloid (Aβ) plaques, primarily composed of the peptide, Aβ, which is liberated from the amyloid precursor protein (APP) via serial cleavage by β- and γ-secretase, (ii) intracellular neurofibrillary tangles (NFTs), comprised of hyperphosphorylated tau (p-tau), and (iii) neuroinflammation including microglial activation and astrogliosis (Hardy and Higgins, 1992; Bertram and Tanzi, 2008). The accumulation of Aβ disrupts normal cell signaling and have downstream effects leading to NFTs, which deprive delivery of nutrients to neurons resulting in cell death, triggering neuroinflammation (Hardy and Higgins, 1992; Bertram and Tanzi, 2008; Kumar et al., 2015; Long and Holtzman, 2019). Figure 1 summaries the key milestones in AD research, drug development, and neurotechnological developments in relation to AD. Recently, several promising diagnostic tools have been developed to aid in the early detection of the disease. However, the therapeutic efforts to treat the disease have only been less fruitful, mostly aimed at managing the symptoms with very little impact on the disease progression itself. Figure 2 summarizes therapeutics aimed at modifying disease pathology or symptoms, along with the optimal time to treat, including presymptomatic prevention.

Traditionally, there are two classes of the United States Food and Drug Administration (FDA) approved clinical treatments for AD: (i) Cholinesterase inhibitors and (ii) *N*-methyl-D-aspartate (NMDA) receptor antagonist (Yiannopoulou and Papageorgiou, 2020). Though these two classes of drugs have shown symptomatic relieve, including improved memory and a decrease in the rate of progression, they have limited efficacy for AD patients as disease modifying drugs. To find more effective treatments for AD, the field has witnessed decades of investigation into the molecular, cellular, biochemical, and genetic factors that contribute to AD development and progression and identified several potential targets (Figure 2). Current targets include APP (O’Brien and Wong, 2011; Muratore et al., 2014; Long et al., 2019; Sawmiller et al., 2019), γ-secretase (GSM) (Wagner et al., 2017), β-secretase (BACE1) (Mullard, 2017; Egan et al., 2018; Panza et al., 2018), Apolipoprotein-E (Sawmiller et al., 2019) and more recently, brain innate immune system targeting neuroinflammation and microglial clearance (Chen M. et al., 2018; Hamblin, 2019; Pan et al., 2019; Wu et al., 2019).

Most AD clinical trials, to date, have targeted Aβ, either by blocking Aβ generation (i.e., with BACE1 inhibitors or γ-secretase inhibitors/modulators) or by directly targeting Aβ aggregation and clearance (i.e., active, or passive immunization of anti-Aβ antibodies) (Sevigny et al., 2016; Mullard, 2017; Egan et al., 2018; van Dyck, 2018). Aβ immunotherapy, e.g., aducanumab, employ antibodies targeting Aβ oligomers and fibrils in the brain. Aducanumab, approved by the FDA in 2021, was first discovered by the Swiss company, neurimmune, inspired by the report of protective human auto-antibodies targeted at cross-linked Aβ oligomers (Moir et al., 2005). Early futility analysis of aducanumab clinical-trial data showed no significant effects on clinical symptoms, including memory loss and disorientation, which halted the phase three aducanumab clinical trial program (Aisen, 2019). However, higher doses of aducanumab given for an extended period (under a trial amendment) reported significant, albeit modest, slowing of cognitive decline in early-stage AD. The aducanumab trial data suggest that treatment should be carried out at the earliest possible stages of AD, or even pre-symptomatically.

Several previous clinical trials targeting Aβ failed often with safety concerns related to safety concerns related to Amyloid Related Imaging Abnormalities vasogenic edemas (ARIA-E) or hemorrhages (ARIA-H), e.g., Aβ immunotherapies-Bapineuzumab, Crenezumab, and Gantenerumab. Meanwhile, the γ-secretase inhibitors, Avagacestat and Semagacestat, worsened symptoms following treatment (Mehta et al., 2017). A more viable approach may involve reducing the generation of longer isoforms of Aβ, e.g., Aβ_42_, which are more prone to amyloid formation can be achieved by modulating the docking site of β-secretase with γ-secretase modulators (GSM), that do not prevent γ-secretase cleavage of other substrates, e.g., NOTCH (Wagner et al., 2017).

The complexity of AD suggests that effective treatment may be a regimen of multiple targets at various stages of the disease. The pathology of AD begins to develop decades before its first clinical symptoms, requiring the application of a strategy of early detection–early intervention. AD neuropathogenesis can be divided into three categories: (i) microscale: genetic mutations and molecular aberrations, (ii) physiological scale: homeostasis, neuroimmunity, and clearance dysfunctions, and (iii) global scale: circuit dysfunction. The pharmacological approach typically targets only one scale. However, a combinatorial approach targeting each of these areas may be required. While this adds another level of complexity in dissecting the pathological, systemic, and molecular changes in AD, it may be one of the best ways to control and stop disease progression.

## Alternatives to Targeting Aβ

In recent years, there have been an increasing number of clinical trials targeting the microtubule-associated protein, tau (LaFerla and Oddo, 2005; Khan et al., 2017; Congdon and Sigurdsson, 2018). Tau is an essential component of the neuronal cytoskeleton, involved with intracellular trafficking and stabilization of microtubules (Wiśniewski et al., 1976; Iqbal et al., 2005, 2010; Gao et al., 2018). Tau pathology (tauopathy) is found in a range of neurological diseases, including progressive supranuclear palsy, traumatic brain injury, stroke, frontotemporal lobe dementia, Pick’s disease, and AD (Iqbal et al., 2005; Spillantini and Goedert, 2013; Gao et al., 2018). Moreover, in AD, Tau and Aβ dysfunction are inextricably linked, with evidence showing tau as an effector downstream of Aβ; (LaFerla and Oddo, 2005; Shipton et al., 2011) other studies demonstrate a feedback loop relationship (Leroy et al., 2012). Recent drug targets focused on tau are aimed at regulating post-translational modifications, such as hyperphosphorylation, acetylation and glycosylation, as well as aggregation (Congdon and Sigurdsson, 2018; Jadhav et al., 2019). These post-translational modifications of tau can improve clearance and inhibit tau accumulation (Jadhav et al., 2019), e.g., using phosphodiesterase inhibitors (Brunden et al., 2009; Long and Holtzman, 2019). Furthermore, methods to directly reduce tau expression using small interfering RNA or antisense oligonucleotides as well as the synthesis of active and passive antibodies against tau are being pursued (Congdon and Sigurdsson, 2018).

While clinical trials based on these targets are currently ongoing, it has become increasingly clear that Aβ and tau pathologies are early stage “initiating” pathologies that begin decades before symptoms and increasingly trigger neuroinflammation, the major contributor to neuronal cell loss in symptomatic patients. The idea that the innate immune response is part of AD pathology first emerged in the 1960s (Akiyama, 1994) and since then, surging evidence have supported this theory (Bronzuoli et al., 2016; Kinney et al., 2018). Based on this theory, reactive gliosis, a phenomenon due to persistent glial activation, leads to cellular dysfunction, neuronal and synaptic loss, and neuroinflammation in response to brain injury (Bronzuoli et al., 2016). Numerous studies have connected innate immunity and neuroinflammation to the AD pathogenesis (Glass et al., 2010; Eldik et al., 2016; Kinney et al., 2018) and the first AD gene involved in innate immunity, CD33, was discovered in family-based associations study in 2008 (Bertram et al., 2008) followed by many more innate immune genes (Bertram and Tanzi, 2019).

Reactive microglia and astrocytes surround both Aβ plaques and neurons dying with tangles, resulting in homeostatic dysfunction and neuronal damage, which in turn can lead to more chronic inflammation and create a devastating feedback cycle (Bronzuoli et al., 2016). Consequently, molecules that can modulate and control the neuroinflammatory process can offer new therapeutic opportunities for AD. One molecule of note being tested to target neuroinflammation implicated in the management of AD is curcumin (Lim et al., 2001; Baum and Ng, 2004; Ringman et al., 2005; Mishra and Palanivelu, 2008; Chen M. et al., 2018). Curcumin has been utilized as a sensitive fluorochrome in combination with a variety of imaging modalities, including two-photon microscopy, positron emission tomography (PET), magnetic resonance imaging (MRI), and near-infrared fluorescence (Cheng et al., 2015). Curcumin has also been shown to suppress inflammatory cascades due to its anti-inflammatory properties (Ringman et al., 2012) via different mechanisms that are beyond the scope of this review. Moreover, the anti-inflammatory properties of curcumin may be able to attenuate AD progression (Lim et al., 2001; Mishra and Palanivelu, 2008; Ringman et al., 2012). There is evidence that curcumin can lower Aβ levels by inhibiting BACE-1 transcription through the activation of the pathway that binds to T cell factor-4, Wnt/β-catenin, a repressor of the BACE1 gene (Chen M. et al., 2018). Due to its high affinity for Aβ, curcumin can also directly inhibit Aβ aggregation *in vitro* and *in vivo* (Chen M. et al., 2018). Though promising, these potential new drugs and targets will require rigorous testing and further investigation prior to their implementation and prescription in the clinic.

**FIGURE 3:**
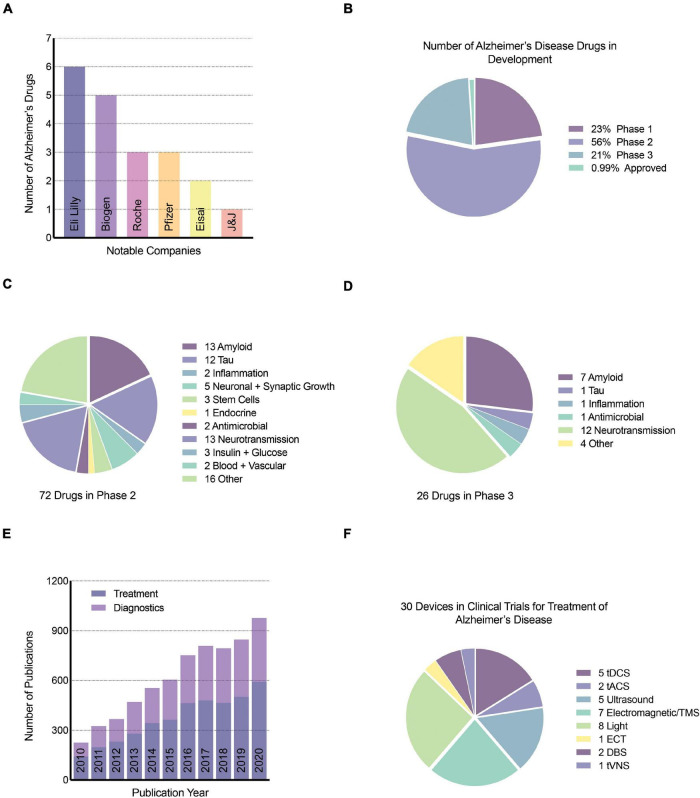
Neurotechnology publications and devices for AD. **(A)** Top notable companies with drugs for AD, **(B)** percentage of AD drugs in phase 1, 2, and 3 of clinical trials, **(C)** number and target of drugs in phase 2 clinical trials, **(D)** number and target of drugs in phase 3 clinical trials, **(E)** number of publications related to the treatment and diagnosis of AD using neurotechnology or stimulation since 2010. Google scholar search keywords used include “neurotechnology,” “Alzheimer’s disease,” and either “diagnostic” or “treatment”. **(F)** The number of brain stimulation medical devices in clinical trials for the treatment of AD was obtained from clinicaltrials.gov, a resource provided by the United States National Library of Medicine. tDCS, transcranial direct current stimulation; tACS, transcranial alternating current stimulation; TMS, transcranial magnetic stimulation; ECT, electroconvulsive therapy; DBS, deep brain stimulation; tVNS, transcutaneous vagus nerve stimulation.

To briefly summarize progress in the treatment of AD in the past decades, it is safe to say that development of AD therapeutics has been extremely challenging (Tolar et al., 2020). Due to the number of the amyloid-based drug therapies that have failed (22.3%) (Liu P.-P. et al., 2019), the amyloid cascade hypothesis has been put into question. It is essential to highlight, though, that most of the failed phase three trial programs involved mild-to-moderate symptomatic AD patients. AD plaque and tangle pathology accumulate in the brain decades before the onset of symptoms, and at this stage of AD pathogenesis, it is mainly neuroinflammation actively driving synaptic and neuronal loss. It would be best to intervene with the initiating pathologies of plaques and tangles as early as possible, in the pre-clinical phase, which might have a better chance of reversing the disease trajectory (Long and Holtzman, 2019). Moreover, for symptomatic patients, neuroinflammation accounts for the majority of the damage at this clinical stage of disease and should be considered the key target (Barroeta-Espar et al., 2019; Elmaleh et al., 2019). Ultimately, the successful treatment and prevention of AD will require both amyloid and non-amyloid strategies, including immunotherapeutics, GSM, anti-tau pathology and tau spreading agents, APOE-based strategies, anti-neuroinflammation, and even anti-microbial approaches (Elmaleh et al., 2019; Long and Holtzman, 2019). The most recent AD drugs in development are summarized in Figure 3.

Finally, it should be noted that developing one drug for AD costs, on average, $5.7 billion (Scott et al., 2014) and usually takes over a decade from the preclinical stage to possible FDA approval. Besides this, there are several challenges, including (i) increasing competition of enrolling patients at early-stage of AD in trials, (ii) functional assessments and cognitive tasks are difficult to measure to achieve robust data, and (iii) human functional assessments cannot be tested in AD animal models (Scearce-Levie et al., 2020). There are also fundamental differences between human and mouse biology, creating challenges to translate science from lab bench to bedside. Thus, it is necessary to leverage other recently evolved *in vitro* models such as three-dimensional (3D) human induced pluripotent stem cell (iPSC)- and embryonic stem cell-derived cultures to replicate the complex environment present in the human brain and accelerate drug discovery (Choi et al., 2014; Kim et al., 2015; Lee H.-K. et al., 2016; Jorfi et al., 2018; Papadimitriou et al., 2018; Park et al., 2018; Kwak et al., 2020; Zhao et al., 2020). These *in vitro* models provide immense value for understanding the underlying mechanisms in AD pathogenesis.

**FIGURE 4:**
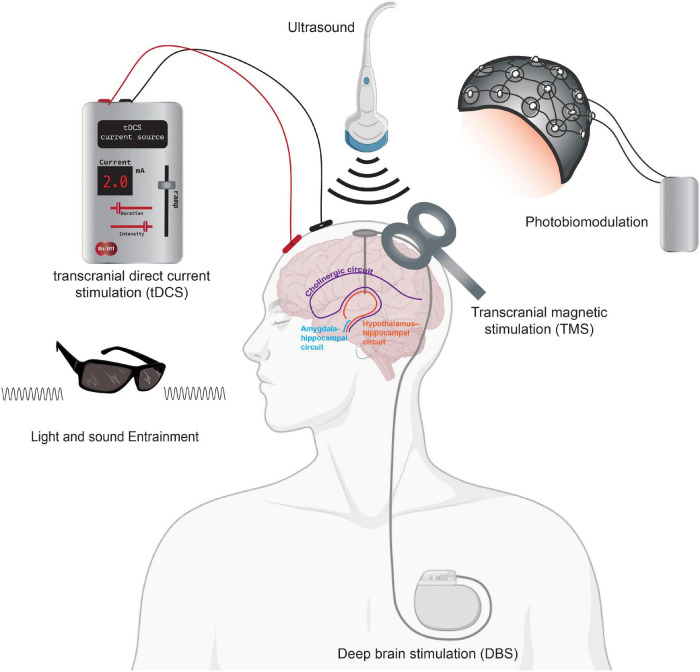
Neurotechnologies using neurostimulation for AD. Summary figure showing tDCS, DBS, TMS, light, and sound modalities as non-invasive neurostimulation treatments for AD therapeutics. Neurostimulation, including deep brain stimulation, can impact brain circuitry including the cholinergic circuit (purple) and the hypothalamus-hippocampal network (orange). Direct stimulation of the amygdala-hippocampal (blue) circuits has also been shown to improve memory in rodent models. Portions of the figure was created with BioRender.com.

In the following sections, we introduce emerging and evolving non-pharmacological neurotechnologies, including light and electrical stimulation aimed at curbing AD pathologies (Figure 3). We highlight the expansion of different non-invasive neuromodulation technologies, as shown in Figure 4, that are starting to be applied as treatment modalities for AD. We focus on the role of magnetic stimulation in mitigating AD pathogenesis. We aim at appraising the extent to which emerging neurotechnologies and non-invasive brain stimulation via different modalities might bring more robust granularity to addressing the clinical complexity of AD and facilitate successful modes of treatment and prevention of this devastating disease.

## Optogenetics and Light Stimulation as an Alzheimer’s Disease Therapeutics

The innovation of optogenetics as a fundamental neuroscience tool highlights how multidisciplinary convergence can truly alter not only the methodologies of a field but the conceptual framework from which to pursue the field. Bacteriorhodopsin had been a critical topic of interest in biology since its discovery in 1971 in the archaeon *Halobacterium salinarum*. In response to green light, this organism pumps protons out of cells (Matsuno-Yagi and Mukohata, 1977) and, in the presence of orange light, pumps chloride inwards (Matsuno-Yagi and Mukohata, 1977; Schobert and Lanyi, 1982). Interestingly, this bacterium lives in high salinity environments where these two-ion transport rhodopsins are crucial for the bioenergetics of the organism (Schobert and Lanyi, 1982). Decades later, light-controlled opsins were adapted to be used for temporal and precise control of neurons, termed optogenetics (Boyden et al., 2005). Optogenetics uses light-gated ion channels from bacteria, or opsins, namely halorhodopsin, channelrhodopsin, and archaerhodopsin, to depolarize or hyperpolarize cell membrane potentials in response to specific wavelengths of light (Boyden, 2011). These proteins can be introduced into cells via transfection of lentivirus or adeno-associated virus packed with the opsin gene (Boyden et al., 2005). Using this method, previous studies have been able to target specific brain regions or cells to artificially modulate cellular activity and behavioral changes (Fenno et al., 2011; Deisseroth, 2015).

More recently, a new method called “GENUS” (Gamma ENtrainment Using Sensory stimuli) was developed that involved exposing mice to light flickering and/or sound at 40 Hz to modulate the activity of multiple brain cell types (Iaccarino et al., 2016; Adaikkan et al., 2019; Martorell et al., 2019). In their first study, Iaccarino et al. (2016) found that optogenetically driving specific neurons, known as fast-spiking parvalbumin-positive (FS-PV)-interneurons, at 40 Hz reduced levels of both Aβ_40_ and Aβ_42_ isoforms in 5XFAD mice. However, this method is limited by the translational feasibility of using optical fiber implants to stimulate the brain. As a potential solution to this technical challenge, the group used visual and audio stimulation at the same gamma frequency to recapitulate the same effect on amyloid load and demonstrated neuroprotection by shifting neurons to a less degenerative state, enhancing synaptic function, augmenting neuroprotective factors, and reducing the inflammatory response in microglia (Iaccarino et al., 2016; Adaikkan et al., 2019; Martorell et al., 2019).

As intriguing as these studies have been, it remains unclear as to how audio and visual stimuli can be propagated across brain regions to achieve effects on Aβ load and neuroprotection. Furthermore, it appears that the effects reported in these studies require long-term treatments and are only transiently detectable subsequent to repetitive GENUS treatment. The authors have reported activation of microglial clearance of Aβ and vascular modulation using GENUS, all of which require independent replication. These studies have not yet addressed how other cell types that are involved in modulating brain oscillations, astrocytes, for example, may be affected by GENUS. Finally, it is also possible that gamma entrainment is fundamentally different across species. So far, negative results have been reported after LED light therapy at 40 Hz for 10 days in patients with prodromal and clinical AD (Ismail et al., 2018). The lack of effects in this study may be confounded by the duration of the treatment and the stage of disease in the recruited patients. Although Iaccarino et al. (2016) reported a significant reduction in Aβ_40_ and Aβ_42_ levels after 7 days of 1-h/day treatments of visual stimulus at 40 Hz in mice, it is likely that human patients require more prolonged treatment to affect amyloid load.

The use of optogenetics to modulate neuronal and network activity, when compared to GENUS, may provide more specificity to different cell types, making it possible to achieve temporal and spatial control. The disadvantage of optogenetics is the invasiveness of the optical fibers required to deliver the light stimulus as well as safety concerns for viral delivery of the opsin genes. While the latter remains a significant challenge, several groups have offered some solutions to enable minimally invasive optogenetics. Chen S. et al. (2018) for example, created molecularly tailored upconversion nanoparticles (UCNPs) that serve as actuators of transcranial near-infrared (NIR) light to stimulate neurons deeper in the brain. This was the first attempt to design a method of optogenetics that did not require an optical cable to be surgically implanted in the brain. However, the efficiency of conversion from NIR to blue light of these nanoparticles is low, which may require a high dose of these particles that would be potentially neurotoxic. Furthermore, NIR light by itself has been shown to affect cognitive function by dissociating nitric oxide to increase mitochondrial membrane potential, thereby increasing ATP production. The increase in nitric oxide can also act as a vasodilator to increase nutrient delivery and metabolite clearance (Purushothuman et al., 2014; Hamblin, 2016, 2019; Hennessy and Hamblin, 2016; Berman et al., 2017, 2019; Saltmarche et al., 2017; Blivet et al., 2018; Salehpour et al., 2018; Chao, 2019; Liebert et al., 2019; Taboada and Hamblin, 2019). NIR light is used in photobiomodulation and can be used independently as a potential treatment for AD. More recently, Gong et al. (2020) created a new opsin with ultra-high light sensitivity (SOUL) that can stimulate macaque cortical neurons using transcranial illumination. SOUL offers the same transcranial stimulation solution without the injection of nanoparticles but affords similar specificity and temporal control as the original optogenetic tool, which proves very promising for stimulating deeper brain regions.

Meanwhile, a multitude of factors including size and flexibility must be considered when designing neural probes/fibers for studying neural function using optogenetics. As a result of the mechanical mismatch between stiff commercial implanted probes/fibers (1–100 GPa) and the soft brain tissue (∼kPa), tissue damage may occur in addition to foreign-body response and eventually glial scarring and interface encapsulation and isolation from the neural cells (Jorfi et al., 2014a; Shoffstall and Capadona, 2018; Frank et al., 2019). Moreover, new innovations in materials development and fibers/probes fabrication will open the door to better understanding of neural processes without compromising the high fidelity of intracortical interfaces in chronic studies (Jorfi et al., 2014b; Park et al., 2019; Guo et al., 2020). More recently, photoresponsive anti-Aβ agents such as ruthenium (II) complex have shown efficacy to inhibit self-assembly of Aβ monomers to reduce cytotoxicity of Aβ aggregates (Son et al., 2018). Increasingly, biocompatible materials such as copper molybdenum sulfide (Jang and Park, 2021) and copper bismuth oxide (Heo et al., 2020) were found to be capable of clearing Aβ aggregates under NIR light, suggesting the potential of combining non-invasive stimulation with stimuli-responsive materials as a therapeutic approach. Taken together, the development of a tool for non-invasive light stimulation has undergone tremendous advances in the past decade. However, optical tools have yet to be developed that can overcome the challenge of having both a non-invasive and a precise tool to selectively stimulate specific brain regions with temporal precision. Incremental advancements are currently paving the way for the potential clinical application of light-based brain stimulation, but much of the mechanism remains unexplored while clinical trials of these non-invasive strategies are ongoing.

## Ultrasound Stimulation

Prior to the deployment of ultrasound as an imaging modality in medicine, it was investigated as a method to stimulate tissues (Baek et al., 2017). In the early 1900s, Harvey explored the use of high-frequency ultrasound waves to change the firing rate of amphibian cardiac muscles (Harvey, 1929). Decades later, Fry et al. (1958) demonstrated, for the first time, the ability to use ultrasound to transiently suppress light-evoked potential in the visual cortex. The potential of ultrasound for brain stimulation has been bolstered by recent technological advancements in focused transducer designs, allowing for more specific targeting at the millimeter scale. In the past decade, the ability of ultrasound to focus on a small region of interest has allowed scientists to explore the application of low-intensity focused ultrasound as a non-invasive neurostimulation tool for disease treatment (Tufail et al., 2011; Baek et al., 2017; Fomenko et al., 2018). Noting the limitation of genetic manipulation in optogenetics, Tufail et al. (2010) investigated the potential of using transcranial pulsed ultrasound to modulate neuronal activity in mice. Following the first demonstration of focused ultrasound to modulate behavior in the awake primate brain, human studies have been initiated (Deffieux et al., 2013). Initial human studies have focused on the primary somatosensory cortex to determine its ability to enhance performance on sensory discrimination tasks (Legon et al., 2014) as well as the primary visual cortex to influence visual and higher-order cognitive functions (Lee W. et al., 2016; Legon et al., 2018). Given the rapid technological improvements over the past decade, it is likely that this modality will overcome spatial resolution challenges.

Ultrasound stimulation is also uniquely positioned to overcome the blood–brain barrier (BBB), which has been a significant obstacle for brain drug development. Magnetic resonance-guided focused ultrasound (MRIgFUS) is an emerging non-invasive modality that can be coupled with microbubbles to transiently open the BBB as a method to reduce plaque burden, trigger neuronal plasticity, and prevent spatial memory deficits (Lipsman et al., 2018). Lipsman et al. (2018) applied this technology to early-stage AD patients. Although no significant therapeutic effect was detected using PET, there were no significant adverse events. Concurrently, Beisteiner et al. (2019) tested the clinical effects of ultrashort ultrasound pulses (transcranial pulse stimulation, TPS) in a small cohort of AD patients and detected no major side effects. They also observed a high level of treatment tolerability with improved neuropsychological scores, which correlated with the upregulation of the memory network based on fMRI imaging, for up to 3 months following treatment.

In addition to using MRIgFUS as a treatment modality, Xhima et al. (2020) successfully used the MRIgFUS method to increase the permeability of the BBB and deliver a TrKa agonist, D3, to the basal forebrain to activate the TrkA-related signaling cascade and enhance cholinergic neurotransmission in mice. This study elucidates a promising direction for the application of MRIgFUS as a delivery tool of molecules that can directly improve neuronal function, which is lost in AD. Furthermore, it overcomes the spatial resolution challenge inherent in ultrasound stimulation by using a targeted approach. Finally, this study highlights the synergistic effect of combining established chemical compounds with non-invasive neurotechnological approaches to enhance the efficacy and safety of AD treatments.

More directly, transcranial ultrasound has been applied to directly modulate amyloid load. Beisteiner et al. (2019) demonstrated improved neuropsychological scores after transcranial pulse stimulation (TPS) using ultrasound lasting up to 3 months with no major side effects. Jeong et al. (2021) also piloted a clinical study of low-intensity transcranial focused ultrasound that Patients demonstrated mild improvement in measures of memory, executive, and global cognitive functions with no reported side effects. Emulating gamma entrainment, Park et al. (2021) utilized 40 Hz ultrasound to reduce Aβ_42_ in the hippocampus and cortex in 5XFAD mice. Given this result, it appears that gamma entrainment can be achieved via different neurostimulation modalities. This might pose ultrasound as the optimal stimulation modality for AD as it can achieve deep and global penetration into the brain that is challenging for light to achieve. Future studies in animal models can confirm the mechanism of action via this modality. Given the non-invasive nature of this method and the lack of side effects observed thus far, it can be more easily tested in humans and achieve easier adoption in the clinic.

Adopting the same conceptual framework from the evolution of light stimulation with biocompatible materials, Jang et al. (2020) were one of the first groups to demonstrate the use of piezoelectric materials for ultrasound-driven dissociation of Alzheimer’s Aβ aggregates. Here, piezoelectric bismuth oxychloride (BiOCl) nanosheets are sono-activated to destabilize and disaggregate Aβ fibrils *in vitro* and in AD mouse brain slices The fundamental characteristics of piezoelectric materials provide many advantages suitable for biomedical applications, particularly in the brain. For example, in addition to its superior electrical power density, these particles can be fabricated below 100 nm to allow for BBB penetration. Upon mechanical stimulation, piezoelectric nanoparticles exhibit a unique effect called piezocatalysis. Piezocatalysis is a new approach to evoke electrochemical reactions via mechanical agitation, such as stirring or vibration, causing charge carriers to transfer from the surfaces of piezoelectric materials to reactants. While the physics is not entirely clear, ultrasound waves can be employed to mechanically interact with piezoelectric nanoparticles to promote generation of electric charges for neuromodulation, drug delivery, and regenerative medicine, and cancer therapeutics (Cafarelli et al., 2021). The coupling of ultrasound with nanobiomaterials with targeted and sono-responsive functionalities has tremendous potential to overcome the limitations of conventional drug delivery systems and treatments (Low et al., 2021).

## Electrical Stimulation

A Roman physician, Scribonius Largus, back in 46 A.D., was first to clinically employ electrical brain stimulation to treat headaches and gout (Kellaway, 1946). Today, using more advanced technologies, we continue to apply the same foundational principle as our ancient counterparts, namely open-loop single-source electrical stimulation. Modern-day deep brain stimulation (DBS) began in 1987 with a preliminary report from a French neurosurgeon, Alim-Louis Benabid (Benabid et al., 1987), who inserted a microwire with four electrical contacts into the thalamus of a patient with tremor and applied high-frequency continuously-on electrical stimulation. During the next few years, Benabid et al. (1991) explored the effects of open-loop high-frequency stimulation on numerous subcortical targets. Experimental data suggested that stimulation of the thalamus was significantly safer than a bilateral thalamotomy (Benabid et al., 1991; Blond and Siegfried, 1991), Similar findings were later reported for pallidal stimulation (Laitinen et al., 1992). The increase in safety over lesion surgeries fueled both the adoption of DBS and the exploration of novel indications. Most immediately, DBS was used for other movement disorders such as bradykinesia, rigidity, and dystonia (Yu and Neimat, 2008). DBS for Essential Tremor was approved by the United States FDA in 1997, for Parkinson’s disease in 2002, and under a humanitarian device exception for Dystonia in 2003. Subsequently, applications of DBS expanded beyond the motor system, including pain, major depression, Tourette syndrome, obesity, anorexia, addiction, epilepsy, pathological aggression, and dementia (Youngerman et al., 2016).

Despite the increasingly successful adoption of DBS as a therapeutic intervention, the underlying mechanisms of action remain poorly understood. Historically, DBS replaced ablative surgery as a safer, more consistent, and reversible alternative. Based on the observed similarity between high-frequency (i.e., >130 Hz) stimulation and surgical lesions on the same brain regions, DBS was assumed to operate as a reversible lesion by inhibiting neuronal cells adjacent to the stimulating electrodes (Herrington et al., 2015). It is now understood that the neuromodulatory effects of DBS are more complex and multifaceted than initially thought. Most studies on the mechanisms of DBS come from non-human primate and human intraoperative neurophysiology studies. From those studies, we know that high-frequency DBS typically produces a local inhibitory effect in basal ganglia targets, which is mediated through several potential mechanisms: sustained depolarization of the neural membrane, inactivation of sodium channels (Beurrier et al., 2001; Magariños-Ascone et al., 2002) and increases in potassium currents (Shin et al., 2007). Additionally, DBS may also act by activating presynaptic terminals on afferents to the soma, creating a local inhibitory or excitatory effect in regions with high inhibitory or excitatory inputs, respectively (Herrington et al., 2015). Moreover, computational models and empirical data have demonstrated that DBS can increase the activity of axons and dendrites, causing downstream regions to experience increases in efferent activity, despite local inhibition at the stimulation site (McIntyre et al., 2004). Finally, DBS is known to induce antidromic activity (Li et al., 2007) causing activation of upstream brain regions (MacKinnon et al., 2005).

Encouraged by the effectiveness of DBS in relieving the symptoms in Parkinson’s patients, recent studies have focused on the use of this technology for AD with some promising results (Laxton et al., 2010; Smith et al., 2012; Kuhn et al., 2014; Sankar et al., 2014; Lv et al., 2018). However, DBS as a treatment is limited by localized inflammation at the site of the implant as well as the precision of the implant for the brain region of interest. The surgical implant of the DBS device can be high risk, and the inflammation caused by the procedure may add to the chronic CNS inflammation in the pathogenesis of AD. However, DBS implants are specific and can help target early AD at the initial insult of the disease. The frequencies of stimulation can also be investigated for modulating immune cell types to target a variety of neural cell types to work concurrently on removing or reducing the pathologies in AD. Furthermore, the implant has the potential to be designed to be both a sensor and stimulator, which opens the door for the development of a closed-loop adaptive system that patients and physicians can monitor from their mobile phones (Vissani et al., 2020). The possibility for such a device paves the way for the development of personalized medicine for the management of neurodegenerative and other neurological diseases (Krauss et al., 2021).

Finally, in a less invasive method, transcranial direct current stimulation (tDCS) can be used to penetrate the skull and modulate neural activity in selected brain regions (Ferrucci et al., 2008; Bystad et al., 2016; Gondard et al., 2019; Liu C. et al., 2019; Turriziani et al., 2019). It is thought that anodal stimulation increases cortical excitability, while cathodal stimulation decreases excitability by influence the cell membrane polarization (Chang et al., 2018). The challenges currently limiting brain stimulation strategies, whether by light, sound, electrical, or by ultrasound (Beisteiner et al., 2019) lie with the lack of understanding of the molecular mechanisms underlying these treatment modalities, and the greater mystery of the complex human brain. Neural activity in the brain generates a series of electrical neural oscillations that specifically contribute to a variety of functions (Osipova et al., 2005; Berridge, 2013; Iaccarino et al., 2016; Martorell et al., 2019; Perotti et al., 2019). External electrical stimulation can be used to mimic those natural oscillations and entrain neurons to better perform functions impaired by neurological disease.

## Electrical Brain Implants

Another emerging area of technological integration with neurotherapeutics lies in electrical brain implants. Recently, a drive toward increasing the capacity of neurotechnological therapies to reach hallmark milestones, such as the neural recording activity with sub-millisecond temporal precision from greater than one-thousand neurons, has stemmed from both United States and global initiatives encouraging the development of next-generation tools to capture more degrees-of-freedom, with higher-throughput. An important driver of this technology draws on the similarities between the relative increase in the recording capacity of neural probes with the increase observed with transistors in integrated circuits (Stevenson and Kording, 2011). Neural probe design has not quite kept pace with this prediction, largely due to fundamental limitation of single shaft microwires, which fail to incorporate into the neural parenchyma over long periods of time, precluding our ability to understand the diversity and variability of neural function over long periods of time required for a truly functional neural prosthetic.

Implantation of electrodes into the brain is a disruptive procedure that results in a local neuroinflammatory and glial response. Numerous factors contribute to the acute response, such as the physical, chemical, and mechanical composition of the electrode as well as features such as the diameter of the probe, shape, and size of the recording tip, cross-sectional area, the implantation procedure itself (Nicolelis et al., 2003; Szarowski et al., 2003; Jorfi et al., 2014a). The acute response typically lasts for up to a week. The chronic response, however, which manifests within the first month following implantation, poses the greatest risk toward the functional longevity of the probe. Neuroinflammatory microglia and reactive astrocytes mount a response against the foreign object and attempt to sequester it from the adjacent brain tissue. A dense encapsulation layer is created through the fusion of multiple macrophages into multi-nucleated cells that ensheath the probe creating a dense barrier between the probe contacts and parenchyma. This functionally increases the electrode impedance and reduces signal-to-noise of the recording implement, inhibits axon growth and probe integration, reduces neural populations near the probe, and may induce secondary injuries through an increasing stiffness-mismatch with adjacent tissue (Biran et al., 2005; Polikov et al., 2005).

Following machined and twisted wire multielectrode, initial designs toward the goal of increasing neural sensing capacity have emerged from advances in microfabrication techniques. The development of Utah and Michigan-type silicon multielectrode arrays have dramatically increased the number of simultaneously sampled sites but remains relatively large and continues to produce a foreign body response reducing longevity (Normann et al., 1999; Takeuchi et al., 2004). Strategies of increasing planar distributions of sensing contacts and monolithic arrangements of multiple Michigan-style silicon probes have further increased neural measuring capacity. Neuropixel (Jun et al., 2017) and Neuroseeker (Raducanu et al., 2017) probes have raised the sensing capacity an order of magnitude above its predecessors. In a recent study, eight Neuropixels were implanted into the mouse cortex allowing researchers to measure from nearly 3,000 densely packed cortical sites (Stringer et al., 2019). The Neuroseeker probe, like the Neuropixel, has the capacity to measure from 1,356 recording sites using temporally resolved multiplexing and is currently the highest density neural probe (Raducanu et al., 2017).

Despite the rapid growth in neural sensing capacity through innovative design and manufacturing, penetrating electrodes continue to suffer from poor long-term stability resulting from the foreign body response. Multidisciplinary research across material science, engineering, and neuroscience has employed numerous strategies to reduce or eliminate the immunological response following electrode implantation [for a complete review, see references (Jorfi et al., 2013; Fattahi et al., 2014; Wellman et al., 2018)]. Biomimicry approaches using surface modifications on the electrode, e.g., through bioactive coatings, biocompatible materials, or drug-releasing materials, have been widely explored. Active molecules, such as nerve growth factor and cell adhesion molecules, have been used in attempts to increase neuron density near the probe surface (Ignatius et al., 1998; He et al., 2006; Kim et al., 2007). Anti-inflammatory compounds and neurotropic media can also be used to reduce immunoreactivity and the number of activated microglia and astrocytes following implants, overall reducing long-term changes in electrode impedance (Taub et al., 2012; Potter et al., 2014; Potter-Baker et al., 2014; Nguyen et al., 2015). Drug-eluting coatings have the advantage of modulating cell behavior at a distance compared with surface coatings. Dexamethasone coatings have been extensively studied and are effective in reducing gliosis and electrode impedance, but long-term efficacy remains unclear (Zhong and Bellamkonda, 2007). Alternatively, *in situ* softening biomaterials inspired by nature have been investigated as substrates for intracortical microelectrode to facilitate ease of implantation into the brain tissue (Capadona et al., 2008, 2012). These smart biologically inspired materials soften significantly upon implantation *in vivo* to better match the mechanical properties of cortical tissue (Harris et al., 2011; Jorfi et al., 2013, 2015; Nguyen et al., 2014, 2015; Potter et al., 2014).

More recently, the development of tissue-like electronics has taken a fundamentally different approach. For example, mesh electronics are designed to remedy the structural, mechanical, and topological mismatch between the brain interfaces and neural substrate allowing for a probe that “looks” and “feels” similar to the human brain. Mesh contains cellular and sub-cellular sized components designed for high-density recording capacity, incorporated into a 3D ultra-flexible scaffold, which allows for interpenetration of cells and diffusion of biochemical species (Hong et al., 2004). Through this unique design, there is little evidence of a long-term immune response, making them indistinguishable from host tissue (Xie et al., 2015). Major breakthroughs have recently been made in this area. Luan et al. (2017) successfully fabricated ultraflexible nanoelectronic thread that allows for integration and glial-scar free neurointegration capability of long-term neural recording.

The rapid development of biocompatible electrical neural probes has clear implications in implantable diagnostic and treatment interventions for a variety of neurological disorders, including Alzheimer’s disease. Once the neuroinflammatory considers are overcome, these implantable allow for real-time diagnostics and treatment delivery. Chae et al. (2021), for example, developed a bimodal neural probe that enables detection of electrical and chemical signals and concurrent drug delivery for neuromodulation *in vivo*. While a variety of form factor considerations will need to be optimized, the current developmental trend has opened up an opportunity for electrical implants to be a novel modality for diagnostics and treatment for neurological disorders with the capability of a closed-loop system personalized to the individual.

## Magnetic Brain Stimulation

In contrast to the previously presented brain stimulation modalities, which require brain implants or other invasive delivery methods, in the last two decades, researchers have turned their attention toward non-invasive magnetic stimulation methodologies (Hallett, 2000). Transcranial magnetic stimulation (TMS) or repetitive transcranial magnetic stimulation (rTMS) has been a long-established investigative tool to explore all areas of cognitive neuroscience (Hallett, 2000). The electrical current in the TMS coils produces changes in the magnetic field, inducing an electrical field sufficient to interrupt regular brain activity. This neurotechnology has been employed to temporarily produce specific neuropsychological effects, e.g., neglect syndrome, visual hallucinations (Hallett, 2000). TMS has also been used to investigate essential cognitive functions such as attention, perception, and learning, and for application in the treatment of movement disorders (Ugawa et al., 2020), epilepsy (Photios et al., 2020), depression (Bozzay et al., 2020), schizophrenia (Vittala et al., 2020) as well as other psychiatric disorders (Hallett, 2000). Despite the plethora of applications and research conducted using TMS, the application of this tool to understand or treat neurological disease is still in its infancy (Oliviero et al., 2015; Turriziani et al., 2019; Baumer and Rotenberg, 2020).

In the context of AD, two studies (Ferreri et al., 2002; Lazzaro et al., 2004) used TMS to demonstrate an increase in motor cortex excitability in AD patients, most likely due to impaired intracortical inhibition in AD patients. Others have used TMS to attempt to improve AD cognitive symptoms such as anomia (Cotelli et al., 2008), comprehension (Cotelli et al., 2010), spatial learning (Wang et al., 2015), and memory (Lee W. et al., 2016). Clinical trials are now underway for testing the effectiveness of TMS as an AD therapeutic, though, its application has been more robustly explored for the treatment of psychiatric diseases, such as depression. More recently, repetitive transcranial magnetic stimulation has been shown to prevent the decline of long-term memories by enhancing the brain drainage system (Lin et al., 2021). This novel mechanism of action for AD therapeutics via magnetic stimulation holds promise for further investigation of this non-invasive method.

One major challenge in TMS treatment has been the ability to fine-tune the magnetic force to a precise location. To tackle this problem, implantation of magnetic coils has been investigated for the potential to activate specific brain regions without having to use a significantly higher magnetic field. These technologies have only recently been used as a tool for neuronal stimulation (Bonmassar et al., 2012, 2018; Park et al., 2013), visual prostheses (Lee S. W. et al., 2016), and other brain-computer interface applications. Furthermore, the technology is limited by materials that are both biocompatible and sensitive to magnetic fields. More recently, Lee et al. (2021) created a magnetic toolkit, m-Torquer, to deliver piconewton-scale forces to neurons to enable remote and consistent neuromodulation in freely moving mice. These efforts demonstrate the push for magnetic neuromodulation in the basic sciences (Ferrucci et al., 2008; Bystad et al., 2016; Chang et al., 2018) and its application in disease pathologies for clinical applications such as neurodegeneration (Falconieri et al., 2019).

## Nanovectors

Nanoscale science has provided an unprecedented degree of control and understanding of the molecular and atomic scales. Their applications have allowed material scientists and chemical engineers to overcome several challenges in drug delivery and medical imaging. For instance, a major challenge in drug delivery has been the lack of specificity of drug distribution to the pathological site of interest, systemic distribution with an inability to concentrate the drug locally, the failure to control the release profile of the drugs, the inability to visualize drug concentration, as well as the undesired side effects associated with systemic delivery of the drug (Mahmoudi et al., 2011). Nanovectors can be made from a range of materials and can be divided into three main categories: lipid-based, non-lipid organic-based, and inorganic (Shen et al., 2012). These nanovectors can be designed to carry a drug payload and decorated with an antibody specific for the site of pathology for active targeting (Shen et al., 2012).

In the past two decades, a few studies have begun to examine the use of nanovectors to deliver therapeutic agents into the AD brain. The first attempts examined the delivery of existing drugs, such as rivastigmine, using poly(n-butyl cyanoacrylate) nanoparticles coated with polysorbate 80 to help overcome the BBB and increase bioavailability (Wilson et al., 2008). Later, direct genetic manipulation was made possible by Alvarez-Erviti et al. (2011), who demonstrated siRNA for BACE1 knockdown, delivery using targeted exosomes. Furthermore, targeted nanoliposomes have emerged as promising and viable delivery system for AD that are biocompatible, tailorable, and have the capability to carry therapeutic molecules, e.g., rivastigmine, curcumin, and aggregation-inhibiting retro-inverted peptides, across the BBB. This is achieved by functionalizing the vector with specific molecules that enable carrier- or receptor-mediated transcytosis (Ross et al., 2018). Combining multiple modifications into a multifunctional liposome is currently a research area of great interest. These studies, however, are limited by the packaged therapeutic molecule therefore constrained by the availability of current effective drugs or known genetic targets.

More recently, nanovectors have also been considered as a direct therapeutic intervention. Jung et al. (2020) for example, constructed Aβ nanodepleters from silica nanostructures that reduced amyloid load in an AD mouse model by 30%. The nanoparticles function by capturing monomeric Aβ while inhibiting Aβ aggregate formation (Jung et al., 2020). This technology aims to reduce Aβ load without the need for any external brain stimulation. However, it is unclear whether the nanodepleters are systemically non-toxic once they capture the Aβ.

Regarding clinical applications, it is unclear if it will be feasible to chronically administer nanomaterial to treat or prevent AD. When considering the pharmacokinetics, a number of factors can affect the biodistribution and half-life, including, but not limited to, material size and composition, the core, surface chemistry (pegylation and surface charge), and ligand functionalization (Haute and Berlin, 2017). Furthermore, accumulation of nanomaterials in the liver and the spleen, as well as other organs, may create safety concerns for translating nanomedicines (Tsoi et al., 2016). This is not only a concern for off-target effects and safety of any nanomedicines but also diminishes the amount of the nanomaterial delivered to the target site for treatment. Several surface modifications have been used to help overcome the challenge of sequestration. While surface chemistry cannot change the probability of cellular interaction, it can affect how long the nanomaterial remains attached to a cell surface and the likelihood that it is internalized (Tsoi et al., 2016). As a result of the uniqueness of each nanoformulation, each therapeutic vehicle and drug combination will need to be comprehensively assessed to better understand its specific pharmacokinetics and address any toxicity potential.

## Magnetic Nanoparticles

Since Freeman et al. (1960) first introduced the use of magnetism in medicine many advances have been made to tailor various magnetic nanoparticles and vectors to achieve target specificity, spatial control, and biocompatibility for the delivery of drugs to minimize side effects. Superparamagnetic iron oxide nanoparticles (SPIONs) are an attractive nanomaterial for developing drug therapy due to their various physical properties, including superparamagnetism, high field irreversibility, high saturation field, and extra anisotropy contributions, to allow magnetic properties only when exposed to an external magnetic field (Mahmoudi et al., 2011). These properties, in combination with the surface chemistry that can be modified for specific functions, has allowed for the use of these particles for controlled drug delivery (Stephan et al., 2010; Mahmoudi et al., 2011; Lee et al., 2013), cell separation (Xu et al., 2011), ion channel control (Huang et al., 2010) and cell control and manipulation (Airan, 2017; Gahl and Kunze, 2018). The flexible surface coating of SPIONs allows active targeting by attaching any functional antibody specific to the site of interest. Active targeting has been a useful feature in the area of neurological disorders as it allows for a method to overcome the BBB (Kaushik et al., 2019). Further modifications, such as that shown by Huang et al. (2016) using Tween80, can also be used to pass through intact rat BBB under the guidance of an external magnetic field. Due to this capability to modify magnetic nanoparticles while also exerting external control, they have been used as a delivery vehicle for currently approved drugs to enhance penetration of the BBB and improve drug distribution into the brain (Baranowska-Wójcik and Szwajgier, 2020; Luo et al., 2020). In the next sections, we will summarize and discuss how the properties of SPIONs have been exploited to create more effective diagnostic and therapeutic platforms for AD, the challenges to clinical translation, and the implications of these nanomaterials for the future of AD treatment.

### Diagnostic Applications of Superparamagnetic Iron Oxide Nanoparticles

**FIGURE 5:**
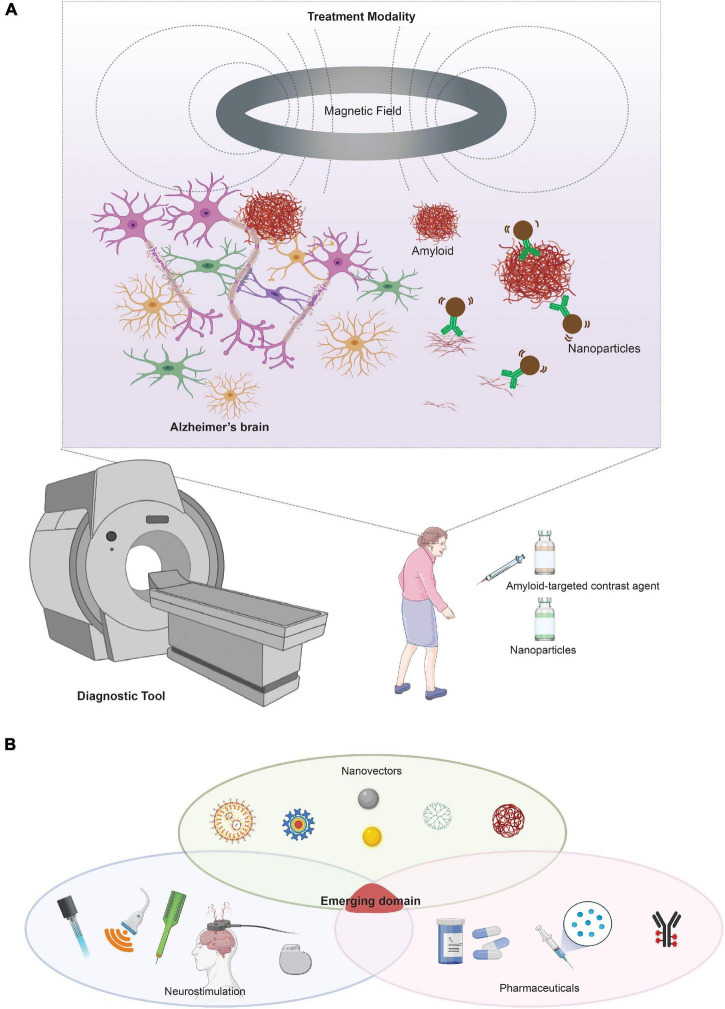
Neurotechnologies based on magnetic fields and magnetic nanoparticles for AD. **(A)** Integration of nanotechnology with neurostimulation for the diagnosis and treatment of AD. Future clinical integration can include nanoparticles as both a diagnostic tool and potential therapeutic modality if integrated with a neurostimulation tool, such as magnetic stimulation. **(B)** Visual representation of an emerging domain integrating neurostimulation, nanovectors, and pharmaceutics/biologics. Portions of the figure was created with BioRender.com.

Due to the biocompatibility of the magnetic nanoparticles and their compatibility with imaging modalities, both magnetic particles and nanoparticles have been extensively explored in the field of AD diagnostics. Traditionally, gadolinium chelates have been used as a paramagnetic contrast agent for T1-weighted MRI imaging. The superparamagnetism of SPIONs enables its use as a novel contrast agent to detect Aβ and tau using T2-weighted magnetic resonance imaging (MRI) as biomarkers for early diagnosis. These nanoparticles have clear advantages in that they have no radiotoxicity, radiopaque, can be modified to be biocompatible, and have specific targets that can visualize Aβ and tau. Recent studies have demonstrated the use of SPIONs to detect amyloid using T2-weighted MRI imaging. More recently, SPIO-PHO was found to cross the BBB to label amyloid plaques in the brain, (Ansciaux et al., 2014) DDNP-SPION nanoparticles injected in a rat AD model (Zhang et al., 2015) while curcumin-conjugated SPIONs were also used to detect amyloid plaque using T2-weighted MRI in mice (Cheng et al., 2015). Since the approval of the Pittsburgh compound (PiB) for AD diagnosis using PET, more work has been done to improve such a diagnostic agent without the need for radiation exposure. In 2018, a modified version of PiB with Mn_0_._6_Zn_0_._4_Fe_2_O_4_, which provided early detection of amyloid plaques using MRI, was reported (Zeng et al., 2018). It is unclear, however, whether injection of the SPION-based imaging contrast has any toxic effects on the disease progression in the long term. Studies have shown Aβ is sensitive to the concentration of the SPIONs, at which a certain threshold can serve to seed more Aβ in the brain and exacerbate disease (Mahmoudi et al., 2013). Nevertheless, SPIONs have entered the clinic as a commonly utilized contrast agent, and its properties potentially allow the integration of diagnostic and therapy in one single visit (Figure 5).

### Therapeutic Applications of Superparamagnetic Iron Oxide Nanoparticles

The possibility of using magnetic nanoparticles to treat or prevent AD has begun to show some promise. A seminal study by Loynachan et al. (2015) demonstrated the feasibility of magnetic particles using their magnetic properties as a treatment to disrupt amyloid aggregates. This is an early proof of concept for the use of alternating magnetic field and nanoparticles to influence AD pathology. While its application in humans remains unexplored, we can envision a synergy with this treatment modality for both diagnostic and therapeutic purposes, as portrayed in Figure 5. There remain, however, two major challenges in the therapeutic application of nanoparticles. First, there is the challenge of crossing the BBB to enable drug delivery or direct therapeutic action. Second, there are limitations to the precision of the spatial control to target specific disease pathologies. To overcome the BBB challenge, recent studies utilized functionalized nanoparticles as drug delivery vehicles to traffic gene therapy and drug molecules across the BBB (Kaushik et al., 2019). To determine if nanoparticles can pass through the BBB while simultaneously delivering gene therapy, Guo et al. (2018) engineered a hybrid siRNA nanoparticle system of targeting Aβ plaques with enhanced BBB penetration. This study demonstrates the versatility of nanoparticles as a vehicle for gene therapy delivery in the brain. Furthermore, Lopez-Barbosa et al. (2020) designed multi-functionalized magnetite nanoparticles for the delivery of siRNA targeted to suppress the expression of BACE1. However, iron oxide nanoparticle delivery of siRNA is limited by the sensitivity of RNA to enzyme degradation and may be more efficacious if encapsulated in a polymeric material.

In terms of specificity, recent approaches have moved away from modifying nanoparticles and expanded to novel methods such as wirelessly manipulating the particles to release drugs or act upon a target. This area is being heavily explored to use magnetothermal tools for precise deep brain stimulation and remote targeted neural control (Cheng et al., 2015; Romero et al., 2016; Rao et al., 2019). The tools are still being developed and tested to enable the magnetothermal stimulation of neural cells. The potential of this technology for non-invasive stimulation of deep brain regions for disease treatment, however, is immense. As the first proof of principle, Kaushik et al. (2019) reported the use of magneto-electric nanocarriers to cross the BBB for the treatment of central nervous system (CNS) diseases (Luo et al., 2020). In this study, they used nanoformulation to create 20-nanometer magneto-electro carriers that can be guided across the BBB on-demand to treat CNS diseases with minimal toxicity to other organs. While further advances have been made to extend this idea, the non-invasive delivery method and specificity required poses a tremendous challenge in terms of bioavailability and toxicity. However, there remain the same challenges as those facing optogenetics, in which a viral infection is required to express the thermal receptors that will respond to the magnetothermal stimulation. To overcome this problem, advances have been made to create a number of stimuli-sensitive nanoparticles for gene delivery, such as focused ultrasound-mediated local drug delivery nanoparticles (Xhima et al., 2020). It is now a high priority to facilitate the convergence of these neurotechnologies to help overcome various challenges in the treatment and delivery of AD therapeutics.

## Quantum Dots

Another important nanomaterial that is shifting from nanotechnology into the health sciences is referred to as “quantum dots.” These semiconductor particles are only a few nanometers in size and have unique optical and electrical properties that have been, most prominently, used in labeling and sensing (Efros, 2019). Interestingly, the use of quantum dots has recently seen a shift from sensing and labeling to acting as a molecular actuator to disrupt the molecular interactions in proteins of disease. While some have explored quantum dots as nanoprobes for the diagnosis of AD, there have been fewer studies of their potential therapeutic applications. Recently, Kim et al. (2018) reported that the use of graphene quantum dots prevents fibrillation of α-synuclein, a major pathological protein in Parkinson’s disease. A similar mechanism could be applied to AD-related pathologies wherein quantum dots might be used to disrupt the hydrophobic interaction of amyloid proteins to prevent their aggregation and accumulation. Gupta et al. (2010) has attempted this using CdSe/ZnS core/shell quantum dots conjugated with biphenyl ethers as both an imaging tool and as a mode of Aβ_42_ disruption via transthyretin inhibition. The authors found effective inhibition of Aβ fibril formation using these quantum dots. This therapeutic direction may sound seemingly promising as it may be able to prevent the initial AD pathological development. However, the *in vivo* applications of quantum dots are limited due to cytotoxicity. Despite this challenge, it has been suggested that cytotoxicity may be reduced by encapsulating quantum dots inside polymers or coating their surface with biocompatible polymers such as polyethylene glycol (Derakhshankhah et al., 2020).

## Challenges of Nanoparticle Technologies

A number of studies have demonstrated promising results for the potential treatment and management of AD using nanocarriers. However, the translation of this technology into the clinic has been slow and sparse. The discrepancy between *in vitro* and *in vivo* results, as well as regulatory barriers, have been significant challenges in advancing nanomedicine beyond the benchtop. Nanotoxicity is a major concern in clinical trials and is dependent on multiple factors ranging from formulation to dosage to cell type. One reason for the discrepant results might be attributed to the adsorption of specific proteins onto the surface of nanoparticles (Derakhshankhah et al., 2020). Upon entrance into a physiological environment, the nanoparticle immediately attracts various proteins and biomolecules to its large surface area, known as the protein corona. The formation of the protein corona can determine the cytotoxicity, bioavailability, and effectiveness of these nanoparticles, which poses a major hurdle in their translation.

In terms of the influence of nanoparticle kinetics on toxicity and potential seeding of Aβ, there are several studies describing these effects. Mahmoudi et al. (2013) found that lower concentrations of SPIONs decreased the rate of Aβ fibrillation rate while higher concentrations had the opposite effect. Later studies confirmed this effect and established the importance of the coating charge on the fibrillation process. It is now known SPIONs with positive coating at lower concentrations can promote fibrillization, while negatively charged nanoparticles can inhibit this process (Mahmoudi et al., 2013). Taken together, these reports underscore the potential to tune these nanoparticles to achieve the desired therapeutic effect without accelerating the fibrillation process.

The benefits of nanoparticles in overcoming the biophysical barriers, such as the BBB, are a major advantage for the future of brain therapeutics (Derakhshankhah et al., 2020). Despite the attractive potential of this method, however, the stability of siRNA and nanoparticles, as well as their controlled and precise delivery, remain challenging. The delivery of gene therapy is also met with low-efficiency transfection rates that may exhibit individual variability. When considering the commercialization of nanotechnology for AD therapeutics, the lack of a reproducible and low-cost method to scale up the production of these materials is another major barrier to overcome for expansive application and commercialization. Furthermore, nanomaterials fall in three FDA regulatory agencies—drugs, devices, and biologics—and, therefore, face more restricted regulatory control.

One of perhaps the most pressing concerns is the safety of these nanoparticles and the safety of their manipulation in the brain. Neurotoxicity of commonly used metal nanoparticles, such as iron oxide and gold nanoparticles, is usually the result of reactive oxygen species that can result in neuronal death or immune cell infiltration (Teleanu et al., 2018). However, these nanoparticles could be made with greater biocompatible and a reduced toxicity by adding surface modifications and a polymer layer. Iron oxide nanoparticles are easily biodegradable *in vivo* as they are added to the iron deposits or incorporated by erythrocytes as part of the hemoglobin (Posadas et al., 2016). As previously mentioned, the size, coating, and concentration may all play a major factor in the potential nanotoxicity, which may require more extensive research and screening as well as a longer development process.

## A Multidisciplinary Perspective

The different stimulation and nanotechnology treatment modalities discussed here possess inherently unique advantages and disadvantages. A growing trend in the neuromodulation field involves examining the combination of multiple brain stimulation tools or using a combination of brain stimulation and nanotechnology tools. For example, Legon et al. (2014) reported on the simultaneous use of FUS and TMS to non-invasively inspect the effect of ultrasound stimulation on neuronal excitability using the motor evoked potential (MEP). The combination of different brain stimulation modalities and drug molecules, however, remains minimally explored. Doxorubicin-loaded microbubbles (Fan et al., 2015) or siRNA-loaded microbubbles (Fan et al., 2016) have been used to improve upon current brain-tumor treatment by employing concurrent MRI and focused-ultrasound. These studies are expanding our concept for what may be possible and how neuromodulation with different modalities could be integrated seamlessly to tackle basic neuroscience questions as well as disease pathologies.

For example, due to the versatility and temporal-spatial precision of optogenetics as a basic neuroscience tool, studies have long sought to overcome the numerous challenges that prevent its translation. First, many of the light-sensitive proteins require wavelengths in the visible light range, which do not sufficiently penetrate tissue or the skull well to reach deeper regions of the brain. Instead, they require an invasive fiber to be implanted to deliver the correct wavelength of light. To address this challenge, material scientists have found that UCNPs may be a promising solution for this challenge. UCNPs are a class of nanoparticles that can absorb tissue-penetrating near-infrared (NIR) light, penetrate more deeply through the skull, and emit wavelength-specific visible light at a localized region to achieve deep brain stimulation. Chen S. et al. (2018) utilized this method to perform optogenetics non-invasively by infecting specific brain regions with ChR and injecting UCNPs in the same region. Subsequently, they stimulated the mice with NIR light to allow the UCNPs to upconvert NIR into blue light, which then will activate the neurons that express ChR. While this is an exciting basic neuroscience direction, a major challenge in translating optogenetics into the clinical is that the use of viral delivery for the opsin gene which has safety concerns for patients. Furthermore, the efficiency of the upconversion is low and may require a large dosage of the nanoparticles for the treatment to have any measurable effects. More recently, to overcome these challenges, Lee et al. (2021) introduced a system, m-Torquer, that employs a genetically encoded mechanosensitive ion channel, Piezo1, in combination with magnetic nanoparticles to enable minimally-invasive modulation of neuronal activation with spatial accuracy. These more recent studies demonstrate the advantage of combining technologies for neuromodulation with those traditional for drug delivery to innovate and uncover more effective, less invasive, and more precise control for not only basic neuroscience research, but also for translational work to pave the path for the future of bioelectronic medicine.

The failed clinical trials for AD drugs, e.g., targeting amyloid pathology, demonstrate a significant gap in our knowledge of the disease process as well as a need for exploring new modalities for AD therapeutics. In comparison, the application of neuromodulatory modalities as disease treatments has been relatively unexplored. The combinatorial effects of existing drugs and biologics with neurotechnologies holds promise to overcome key challenges in the brain therapeutics space. So far, significant effort has been placed on both understanding the mechanism and on exploring the treatment protocols for AD in the modalities of light and electrical stimulation, but little is known regarding the other modalities. A number of these neurotechnologies focus on the mechanical manipulation of AD pathologies or activating a molecular pathway that leads to either the clearance of amyloid or attenuation of the production of the pathological amyloid species. The combination of different brain stimulation modalities and drug molecules, however, remains to be explored. While each technological domain discussed in this review can be developed into some form of treatment on its own, the current trend suggests that several independent technologies may need to converge if we are to overcome the various challenges in neurological disease therapeutics and implement them as effective therapies for AD. Hence, there is a particular need for scientists, clinicians, and engineers with complementary areas of expertise to collaborate in a transdisciplinary manner to harness the power of genetics, cellular and molecular biology, chemistry, pharmacology, surgery, bioengineering, material science, optics, electronics, data science, and various clinical specialties.

While the role of stimulation modalities for the diagnosis, treatment, and prevention of AD are still in their infancy, it is certain that future developments in neurotechnologies will need to move beyond merely the direct modulation of neurons to explore other targets, such as microglia, astrocytes, the meningeal lymphatics, the neurovascular unit, neural oscillations, and the peripheral immune system. The intersection of nanotechnology and AD will hopefully lead to more effective treatment modalities and tools for a number of neurological disorders. As more evidence accumulates to connect lifestyle and comorbidities with AD, such as lipidemia (Wood et al., 2014), gut dysbiosis (Jiang et al., 2017), diabetes (Li et al., 2015), and hypertension (Gabin et al., 2017) future modes of AD treatment and prevention may need to target both specific AD pathologies and generalized disease pathways, such as inflammation, cholesterol, and blood pressure to best manage the disease and ensure the best quality of life for patients. Finally, neurotechnologies need to be tailored to targeting disease progression at the correct stage of the disease, beginning pre-symptomatically, with effective secondary prevention onto acute treatment.

## Author Contributions

MJ proposed and guided the direction of the manuscript. SN, MJ, and SRP wrote the main body of this manuscript. RET and DYK provided supervision and contributed to the concept, design, and writing of the review. All authors contributed to the article and approved the submitted version.

## Conflict of Interest

The authors declare that the research was conducted in the absence of any commercial or financial relationships that could be construed as a potential conflict of interest.

## Publisher’s Note

All claims expressed in this article are solely those of the authors and do not necessarily represent those of their affiliated organizations, or those of the publisher, the editors and the reviewers. Any product that may be evaluated in this article, or claim that may be made by its manufacturer, is not guaranteed or endorsed by the publisher.
