# Supplementary material for: Neurotechnological Approaches to the Diagnosis and Treatment of Alzheimer’s Disease
Source: Front Neurosci. 2022 Mar 24;16:854992. doi: 10.3389/fnins.2022.854992 (PMC8989850; doi:10.3389/fnins.2022.854992)
Supplement: Supplementary file 1 [file Data_Sheet_1.pdf]

Sources for the images used in Figure 1.

1906 – https://simple.wikipedia.org/wiki/File:Alois_Alzheimer_002.jpg
The National Library of Medicine.

1966 – https://physoc.onlinelibrary.wiley.com/doi/abs/10.1113/jphysiol.1966.sp007902
Krnjević K, Randić M, Straughan DW. An inhibitory process in the cerebral cortex. J Physiol. 1966;184(1):16-48. doi:10.1113/jphysiol.1966.sp007902

1974 – https://academic-oup-com.ezproxy.bu.edu/jnen/article/33/1/98/2612750
Powell HC, London GW, Lampert PW. Neurofibrillary tangles in progressive supranuclear palsy. Electron microscopic observations. J Neuropathol Exp Neurol. 1974 Jan;33(1):98-106. doi: 10.1097/00005072-197401000-00007. PMID: 4812327.

1984 –
https://www.sciencedirect.com/science/article/abs/pii/S0006291X84801904?via%3Dihub
Glenner GG, Wong CW. Alzheimer's disease: initial report of the purification and characterization of a novel cerebrovascular amyloid protein. 1984. Biochem Biophys Res Commun. 2012 Aug 31;425(3):534-9. doi: 10.1016/j.bbrc.2012.08.020. PMID: 22925670.

1985 –
https://www.researchgate.net/publication/304134458_Overview_of_Transcranial_Magnetic_Stimulation_History_Mechanisms_Physics_and_Safety
George, M. S., & Belmaker, R. H. (Eds.). (2007). Transcranial Magnetic Stimulation in Clinical Psychiatry. American Psychiatric Publishing, Inc..

2001 – https://www.ncbi.nlm.nih.gov/pmc/articles/PMC6763047/
Agdeppa ED, Kepe V, Liu J, et al. Binding characteristics of radiofluorinated 6-dialkylamino-2-naphthylethylidene derivatives as positron emission tomography imaging probes for beta-amyloid plaques in Alzheimer's disease. *J Neurosci*. 2001;21(24):RC189. doi:10.1523/JNEUROSCI.21-24-j0004.2001

2004 – https://onlinelibrary.wiley.com/doi/10.1002/ana.20009
Klunk WE, Engler H, Nordberg A, Wang Y, Blomqvist G, Holt DP, Bergström M, Savitcheva I, Huang GF, Estrada S, Ausén B, Debnath ML, Barletta J, Price JC, Sandell J, Lopresti BJ, Wall A, Koivisto P, Antoni G, Mathis CA, Långström B. Imaging brain amyloid in Alzheimer's disease with Pittsburgh Compound-B. Ann Neurol. 2004 Mar;55(3):306-19. doi: 10.1002/ana.20009. PMID: 14991808.

2010 – https://onlinelibrary-wiley-com.ezproxy.bu.edu/doi/full/10.1002/ana.22089
Laxton AW, Tang-Wai DF, McAndrews MP, Zumsteg D, Wennberg R, Keren R, Wherrett J, Naglie G, Hamani C, Smith GS, Lozano AM. A phase I trial of deep brain stimulation of memory circuits in Alzheimer's disease. Ann Neurol. 2010 Oct;68(4):521-34. doi: 10.1002/ana.22089. PMID: 20687206.

2016 – https://www.nature.com/articles/nature20587
Iaccarino HF, Singer AC, Martorell AJ, Rudenko A, Gao F, Gillingham TZ, Mathys H, Seo J, Kritskiy O, Abdurrob F, Adaikkan C, Canter RG, Rueda R, Brown EN, Boyden ES, Tsai LH. Gamma frequency entrainment attenuates amyloid load and modifies microglia. Nature. 2016 Dec 7;540(7632):230-235. doi: 10.1038/nature20587. Erratum in: Nature. 2018 Oct;562(7725):E1. PMID: 27929004; PMCID: PMC5656389.

2018 – https://www.nature.com/articles/s41467-018-04529-6
Lipsman N, Meng Y, Bethune AJ, Huang Y, Lam B, Masellis M, Herrmann N, Heyn C, Aubert I, Boutet A, Smith GS, Hynynen K, Black SE. Blood-brain barrier opening in Alzheimer's disease using MR-guided focused ultrasound. Nat Commun. 2018 Jul 25;9(1):2336. doi: 10.1038/s41467-018-04529-6. PMID: 30046032; PMCID: PMC6060168.
